# Prolonged Ocular Foreign Body Found on Repeat Visit to a Second Emergency Department

**DOI:** 10.7759/cureus.37819

**Published:** 2023-04-19

**Authors:** Sara Safari, Conor J McLaughlin, Avani Shah, Bryan G Kane

**Affiliations:** 1 Department of Emergency and Hospital Medicine, Lehigh Valley Health Network/University of South Florida (USF) Morsani College of Medicine, Allentown, USA; 2 Department of Surgery, Division of Ophthalmology, Lehigh Valley Health Network/University of South Florida (USF) Morsani College of Medicine, Allentown, USA

**Keywords:** social determinants, visual acuity, translucent object, corneal abrasion, intraocular foreign body

## Abstract

We describe a case where the patient presented to the emergency department (ED) with ocular irritation in the right eye with concomitant blurry vision that had been persistent for a week. The cause of this patient’s ocular irritation and worsening visual acuity was determined to be a retained foreign body of the limbus. The foreign body had been in the patient’s eye for about four months before he began to experience these symptoms. The four-month duration was established based on initial symptoms and a prior ED visit with no noted eye injury or foreign body detection, as well as the degree of overlying epithelization. This case highlights the importance of obtaining a thorough history and physical examination while emphasizing the high index of suspicion needed for translucent foreign bodies. Here, an inert foreign body erupted four months after injury. Additionally, this case stresses the importance of transition of care for ophthalmologic conditions. Consideration of any social determinants of health that could prevent as an example.

## Introduction

Ocular injuries are common reasons patients present to the emergency department (ED) [1]. Of these ocular injuries, one of the most common diagnoses of blunt injury is corneal abrasions [2]. Corneal abrasions typically present with acute onset ocular pain, tearing, and photophobia where the patient likely recalls recent ocular trauma history [3]. The main goal in treating ocular trauma is to preserve the integrity of the globe [1]. If there is an intraocular foreign body present, like wood or metal, they should be removed by an ophthalmologist as they are reactive and can lead to irreversible damage [1]. Glass and plastic foreign bodies may be retained as they are inert, but the patient must be followed up as an outpatient [4]. In all cases involving ocular injuries, prompt recognition and management are necessary to preserve ocular function [1]. 

Here we describe a unique presentation of a young man who had a chief complaint of eye irritation with no clear inciting event and who presented with one week of escalating symptoms. During the ED evaluation of the eye, abnormalities were noted in the limbus. With further slit-lamp examination, a retained foreign body of the corneal limbus was found that was missed on an initial ED visit four months prior.

## Case presentation

A 28-year-old male patient with no reported past medical history presented to the ED with a chief complaint of right eye irritation. The patient reported that he began to experience right eye irritation approximately one week prior to evaluation with no known insult to the affected eye. He noted he was currently working as a temporary contracted forklift diver but denied any work or non-work-related trauma, environmental, chemical, or allergen exposures since arriving to the area one month prior to complaint. He describes the irritation starting as a severe, central pain that caused tearing for the first three to four days, then progressed to a constant burning sensation with concurrent conjunctivitis. The patient also denied fever, chills, headaches, visual field deficits, ocular motility defects, mucoid/purulent discharge, nasal drainage, sore throat, oral swelling, ear pain or discharge, changes in hearing, numbness or tingling of the face/neck, or abnormal skin changes. He decided to come to the ED for evaluation because of new-onset blurry vision in the right eye and the persistent pain. He has no history of use of corrective lenses including contact lenses The patient’s right eye demonstrated significant conjunctival injection. His pupils were equal and appropriately reactive to light. The rest of the patient’s examination was within normal limits. Visual acuity was 20/20 in the left eye and 20/30 in the right.

The patient was given 0.5% proparacaine ophthalmic solution and a bedside slit lamp examination was performed. During examination, an abnormal reflection of light was noticed between the 6 and 7 o’clock positions of the corneal limbus. The anterior chamber of the right appeared hazy, but no distinct cells and flare were appreciated. Abnormal light reflection was noted. Fluorescein stain test was performed which revealed a negative Seidel’s test but positive uptake in the 3 o’clock position. Due to the abnormal reflective findings raising concern for possible uveitis, poor historical correlation to the abrasion findings, and multiple social barriers to close outpatient follow-up ophthalmology such as access to transportation and knowledge of the local area was called to the bedside for further evaluation to determine the need for topical steroids.

**Figure 1:**
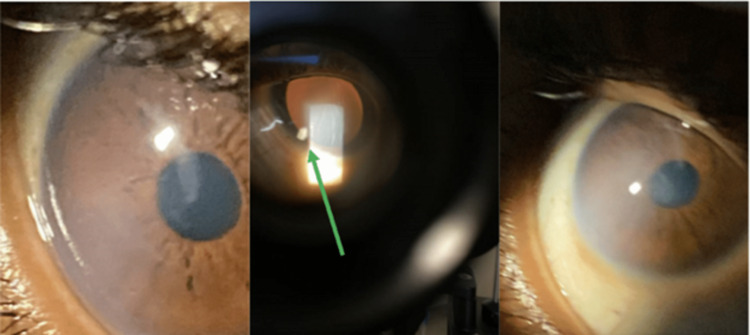
Reflective phenomenon of eye. Middle image shows the glass fragment with higher magnification, denoted by the green arrow, measuring 1.7mm x 1.2mm with irregular borders.

Upon further examination with higher magnification, the ophthalmologist determined the reflective phenomenon was due to an embedded suspected glass fragment measuring approximately 1.7mm x 1.2mm with irregular borders (Figure 1). The ED noted anterior chamber haziness, consistent with the evidence of prior scaring found. These recurrent symptoms were felt to indicate the glass fragment had recently partially erupted. Upon further discussion with the patient, he remembered his exposure to glass shards approximately four months prior to evaluation. He revealed his cousin’s car had been involved in a crash in which the windshield had shattered. A few days afterwards, the patient was in the car, and he remembered noticing a few fine particles on his hands that day and feels he may have rubbed his eye prior to cleaning it off. He notes he did have some irritation then, but was told of no foreign bodies in his eye after being evaluated by a local ophthalmologist in Texas. The irritation spontaneously resolved without other problems until this recent complaint. It is interesting to note that the windshield had already been replaced when he was in the vehicle.

In this patient with a retained foreign body of the corneal limbus, moxifloxacin ophthalmic drops four times a day, prednisolone drops four times a day, and atropine 1% drops twice a day were started. Additionally, once inflammation was improved, there was a plan for surgical removal of the glass body. Unfortunately, despite best efforts, the patient did not follow up with ophthalmology for that surgery.

## Discussion

This case is an important topic for discussion because of how dangerous foreign bodies can be for a patient's health and vision [5]. A high index of suspicion must be maintained for foreign bodies which are translucent [1]. If left untreated, foreign bodies of the cornea/conjunctiva can lead to numerous complications including infection, corneal abrasions/ulcers, uveitis, cornea/globe rupture, corneal scarring, and permanent vision changes/loss [1,6]. Therefore, in cases of ocular foreign bodies, it is pertinent to quickly identify and remove the findings to minimize potential damage. Treatment with appropriate antibiotic prophylaxis is a mainstay of ED management, as is prompt ophthalmology follow-up [6-8]. Evaluation for possible globe rupture with a fluorescein stain test with a Wood's lamp remains a critical action, prompting emergent ophthalmology evaluation [8,9]. If there is concern for an intraocular foreign body, imaging of the globe should be pursued for further evaluation [8]. Imaging may include orbital x-rays, computed tomography scan, or ultrasound depending on the composition of the foreign body [10]. For glass, each of these imaging techniques has various sensitivities reported in the literature [11]. Magnetic resonance imaging is to be avoided for any potentially metallic foreign body.

In terms of the patient’s history, it is atypical for a simple corneal abrasion to both have no clear inciting event or to present with persistent symptoms for one week. This case therefore highlights the importance of a full ocular evaluation, including fluorescein staining and slit lamp examination [10,12]. Here, the initial examination with a Wood’s lamp was unable to appreciate the abnormal findings of the corneal limbus later seen with the aid of the slit lamp. The re-eruption of a foreign body occurring after months, as took place in this patient, is uncommon. Given the inert nature of glass, it has been suggested that absent the evidence of infection, those foreign bodies may be allowed to remain in the cornea and followed serially [12]. Reports of retained glass foreign bodies include a case reporting eruption 21 years after injury [13]. The cornea is composed of five layers, with the epithelium most anterior [14]. This epithelium is replaced, on average, every two weeks, and when disrupted causes pain [14]. It is most likely therefore that glass in this case was in a deeper layer and in the week prior to presentation it began erupting through the patient’s corneal epithelium. The literature notes a single case of the spontaneous extrusion of an eyelash that became embedded in the cornea, pulled into the eye by a pellet fired from a shotgun [15]. The authors felt that “over time, the hair was extruded backwards along its original course of entry” [15]. Based on our patient’s presentation, a similar eruption occurred here.

Healthcare coordination is critical to the effective management of ophthalmologic emergencies [10,12]. In our case, the patient presented to an ED, so consideration of Emergency Medicine Treatment and Labor Act (EMTALA) and its unique application to ocular emergencies is required. EMTALA does recognize the equipment limitations that may exist in an ED, so an overt discussion of the risks and benefits of transferring the patient to an “eye clinic” or outpatient office must occur [16]. In addition to ophthalmologic equipment, the need for imaging as discussed above and the possibility of operative intervention are considerations. In our patient’s case, the decision was made collaboratively to evaluate in the ED. Part of that decision included consideration of the patient’s access to transportation and recent relocation, both of which have been described as social determinants which may impact the outcome for ocular foreign bodies [17,18]. These issues may have contributed to this patient being lost to follow-up for his surgery.

## Conclusions

This case highlights the importance of obtaining a thorough history and physical examination while emphasizing the high index of suspicion needed for detecting translucent ocular foreign bodies. The glass foreign body went undetected after an ED visit four months prior to the onset of the presenting symptoms, likely due to the size and transparency of the fragment. The prompt consultation with ophthalmology allowed for the use of specialized magnification instrumentation to identify the abnormality and further history clarification. Given the complexity of management of ocular foreign bodies, awareness of any barriers to care is an additional consideration.
